# When the inner clock fades: Interoceptive decline and consolidation of phase resetting in cortical rhythms by cardiac events underlie healthy lifespan aging

**DOI:** 10.1162/IMAG.a.1319

**Published:** 2026-07-29

**Authors:** Kirti Saluja, Dipanjan Roy, Arpan Banerjee

**Affiliations:** National Brain Research Centre, Manesar, India; Centre for Brain Science and Applications (CBSA), School of AIDE, Indian Institute of Technology, Jodhpur, India

**Keywords:** interoception, resting state, time perception, heartbeat evoked responses (HERs), Granger causality (GC), inter-trial phase coherence (ITPC)

## Abstract

The brain continuously integrates signals from the external environment along with internal bodily cues to support adaptive cognition and homeostasis. Among these, multiple visceral rhythms, such as respiration, circadian fluctuations, and cardiac activity, contribute to the organization of intrinsic brain dynamics. The present study focuses on cardiac signals, as the heartbeat provides a continuous, quasi-periodic physiological rhythm that enables precise time-locking of neural responses and offers a tractable model for investigating brain–body interactions at rest. Interoceptive processing is influenced by factors, including arousal, emotional state, and aging, and is known to be altered in neurological conditions such as Parkinson’s disease and dementia. Here, we investigated how the cortical representation of cardiac signals changes across the adult lifespan and the mechanisms underlying this reorganization. Using a large human cohort (N = 620, aged 18–88 years) with simultaneous resting-state MEG and ECG, the study found that cortical heartbeat-evoked responses (HERs) showed distinct amplitude changes in the 180–320 ms window post the heartbeat including a systematic reduction in amplitude with age. Cardiac signals modulated the phase of ongoing theta-band neural oscillations rather than altering overall power, and this phase-resetting effect became more consolidated in older adults. Source analysis revealed that resting-state HERs originated primarily from fronto-temporal regions, including orbitofrontal, frontal, and temporal pole areas, and exhibited a clear age-related shift from predominantly frontal to more temporal generators. Directed connectivity analyses revealed an age-related shift in heart–brain communication, characterized by increased heart-to-brain and decreased brain-to-heart Granger causality. Together, these findings demonstrate that cardiac processing undergoes systematic age-related reorganization, characterized by changes in response amplitude, phase dynamics, and heart–brain interactions. These alterations may contribute to broader age-related differences in cognitive functions such as attention, emotions, and time perception.

## 1 Introduction

The integration of internal bodily signals with ongoing neural activity is fundamental to adaptive brain function and the maintenance of homeostasis. Among these signals, cardiac activity provides a continuous, quasi-periodic interoceptive input that interacts with intrinsic neural dynamics. Rather than functioning as a dedicated “internal clock” (Sadeghi et al., 2023), it is increasingly conceptualized as a temporally structured physiological rhythm that shapes cortical activity and contributes to perception, attention, and memory processes (Arslanova et al., 2023, Khoshnoud et al., 2024) making it a tractable model for studying brain–body interactions.

Aging is accompanied by widespread changes in cognition and brain function, including alterations in attention, emotion processing and subjective experience of time, together with large-scale changes in neural oscillatory activity and functional connectivity. Although these changes are well documented, the mechanisms underlying their emergence remain incompletely understood. Emerging evidence suggests that they may partly reflect alterations in the integration of internal bodily signals with ongoing neural activity (Azzalini et al., 2019). For example, neural responses to individual heartbeats modulate conscious visual perception, demonstrating that cardiac signals can shape ongoing cortical activity and behavior (Park et al., 2014). Likewise, interoceptive awareness has been linked to the subjective experience of time, with individuals exhibiting greater sensitivity to bodily signals showing stronger alterations in perceived time passage (Droit-Volet et al., 2025; Sadeghi et al., 2023). Interestingly, subjective time perception itself changes across the lifespan, with many individuals reporting an apparent acceleration of time with advancing age (Friedman & Janssen, 2010), a phenomenon also observed in neurodegenerative conditions such as Parkinson’s disease (Terao et al., 2021) and dementia (Haj, 2016). Together, these findings raise an important question: how is the cortical representation of cardiac activity reorganized across the adult lifespan during the resting state, and what neural mechanisms underlie this reorganization?

One promising avenue for investigating this interaction involves heartbeat-evoked responses (HERs), which reflects the brain’s response to afferent cardiac signals (Craig, 2002; Critchley et al., 2004; Garfinkel et al., 2014; Kim & Jeong, 2019; Wittmann & Meissner, 2019). Cardiac signals reach the insula, Anterior cingulate cortex (ACC), Orbitofrontal cortex (OFC), and related regions via vagal and spinal pathways (Thayer & Lane, 2009). HERs have been implicated in a wide range of cognitive and affective processes, including arousal (Luft & Bhattacharya, 2015), predictive coding (Banellis & Cruse, 2020; Park et al., 2014), interoceptive accuracy in emotional tasks (Garfinkel et al., 2015; Pollatos & Schandry, 2004), heart disease (S. S. Khalsa & Verdonk, 2022), somatosensory perception (Al et al., 2020), attention (Petzschner et al., 2019), consciousness (Babo-Rebelo et al., 2016), mental disorders (Schulz et al., 2015; Terhaar et al., 2012; Treves, 2020), time perception (Khoshnoud et al., 2024), and, importantly, aging (Kamp et al., 2021). Importantly, HERs provide a neurophysiological index of interoceptive processing, rather than a direct measure of interoceptive accuracy or subjective awareness.

Despite this, most HER studies have focused on task-dependent paradigms, typically examining how specific phases of the cardiac cycle influence perception or behavior. This emphasis limits insight into endogenous, resting-state processing of cardiac signals, which may play a fundamental role in shaping intrinsic brain dynamics. Prior work has shown that heartbeat-related activity can synchronize cortical theta oscillations at rest, forming large-scale networks linked to interoception and emotion (Kim & Jeong, 2019). Such phase-based mechanisms may provide a means by which cardiac signals temporally organize neural activity; however, their role in aging remains unclear. In general, evidence for age-related changes in HERs is limited. For example, (Kamp et al., 2021) reported age-related differences in HER amplitude associated with metacognitive performance, although this work was based on relatively restricted age ranges i.e. (N = 49 older adults, 63–82 years; N = 28 young adults, 20–36 years). More broadly, aging is known to influence both brain structure and function (Sahoo et al., 2020), yet it remains unclear how cortical representations of cardiac signals, and their underlying network dynamics, are reorganized across the adult lifespan. In particular, whether age-related changes are reflected in HER amplitude, phase dynamics, or directed interactions between cardiac and cortical systems remains largely unexplored.

In the present study, these gaps are addressed using resting-state magnetoencephalography (MEG) data from a large lifespan cohort (N = 620, aged 18–88 years). Age-related changes in the neural processing of cardiac signals are investigated by examining heartbeat-evoked responses derived from simultaneous MEG and ECG recordings at both the sensor and the source levels. Specifically, we investigated age-related changes in heart-brain coupling at three complementary levels. First, we quantified heartbeat-evoked responses (HERs) to determine whether cortical responses to cardiac events vary across the lifespan. Second, we examined heartbeat related neural dynamics using inter-trial phase coherence (ITPC) to determine whether aging alters the phase organization of the ongoing cortical activity in response to the cardiac events. Third, we characterized the directional organization of heart-brain interactions using Granger Causality (Barnett & Seth, 2014; Dhamala et al., 2008; Granger, 1969), testing whether heart-to brain, brain to heart, and cortico-cortical interactions are reorganized with age. Together, these analyses aim to characterize how interoceptive processing and intrinsic heart–brain interactions are systematically reorganized across the adult lifespan, providing a neurophysiological framework for future studies investigating their relationship with age-related changes in cognition and behaviour.

## 2 Materials and Methods

### 2.1 Dataset and participants

The Cambridge Ageing Neuroscience cohort (Cam-CAN) is a large-scale, multimodal, cross-sectional, population based lifespan (18–88 years) study (Shafto et al., 2014; Taylor et al., 2017). The Cam-CAN project involved two stages. During the first stage, cognitive assessments and tests for hearing, vision, balance, and speeded response, were conducted on 2681 participants at their homes. The data collected during stage 1 was also used to screen participants for stage 2. Those with poor hearing, poor vision, neurologic diseases (such as stroke or epilepsy), or a score of less than 25 in the MMSE cognitive assessment examination were excluded from further participation. This study was conducted in accordance with the Helsinki Declaration, and received approval from local ethics committee, Cambridgeshire 2 Research Ethics Committee (reference: 10/H0308/50).

In the second stage of the project, 700 participants (50 men and 50 women from each age band) were screened and recruited to undergo testing at the Medical Research Council (United Kingdom) Cognition and Brain Sciences Unit (MRC-CBSU) in Cambridge. Participants underwent MRI scans, MEG recordings, and completed various psychological tests and neuroimaging assessments during this stage. In the current investigation, only resting-state MEG data were included for analysis, which was acquired concurrently with ECG recordings. Moreover, given that the primary objective of the present study is to examine the cortical representation of the heartbeat across different age groups, the participants were chosen thorough a screening process for cardiovascular disorders and associates disorders. Those who had history of uncontrolled high blood pressure, stroke or undergone any heart operation or used any blood vessel device/pacemaker were subsequently excluded from the study cohort.

In the present investigation, continuous and categorical approaches were employed to comprehensively capture age-related functional differences. For the continuous analysis, each participant’s age was treated as a single data point. Conversely, for the categorical analysis, the participants were segregated into three distinct groups based on their age: Young adults (18–40 years), Middle adults (41–60 years) and Older adults (61–87 years) as outlined in Table 1.

**Table 1.** Demographic information of Cam-CAN neuroimaging cohort.

Measure	Young adults	Middle adults	Older adults
N	177	198	245
Age range (years)	18–40	41–60	61–87
Mean age ± standard deviation (years)	31.32 ± 6.04	50.28 ± 5.64	72.88 ± 7.27
Female %	48.02	51.01	48.39

### 2.2 Data acquisition

#### 2.2.1 MEG resting state

The MEG data utilized in this study were obtained from the publicly available Cam-CAN repository, accessible at http://www.mrc-cbu.cam.ac.uk/datasets/camcan/. For all the participants, MEG data were acquired by Elekta Neuromag, Helsinki, using a total of 306 channels, including 102 magnetometers and 204 orthogonal planar gradiometers. The data were collected in a magnetically shielded room (MSR) with low lighting. Data sampling was performed at 1000 Hz with a high-pass filter of 0.03 Hz. Head-position indicator (HPI) coils were used to continuously monitor the position of the participant’s head within the MEG helmet. Additional electrodes were used to record horizontal and vertical electrooculogram (EOG) signals to monitor eye blinks and movements. The acquisition of eyes-closed resting-state MEG data was carried out for at least 8 minutes and 40 seconds.

#### 2.2.2 ECG resting state

ECG data were recorded concurrently with MEG acquisition using a pair of bipolar electrodes with a sampling rate of 1000 Hz. The acquisition lasted at least 8 minutes and 40 seconds.

### 2.3 Data preprocessing

The Cam-CAN provided the MEG processed data, which underwent a pre-processing pipeline that involved temporal signal space separation to eliminate noise from HPI coils, environmental sources, and continuous head motion correction. A max filter was used to remove the main frequency noise (50-Hz notch filter) and reconstruct any noisy channel. Further information on data acquisition and preprocessing can be found at (Shafto et al., 2014; Taylor et al., 2017). The MEG time series was detrended, and a bandpass filter was applied with a 1–45 Hz range. Further, ICA (Independent Component Analysis) was employed in MNE-Python to eliminate artifacts such as eye blink and cardiac field.

**Fig. 1.**
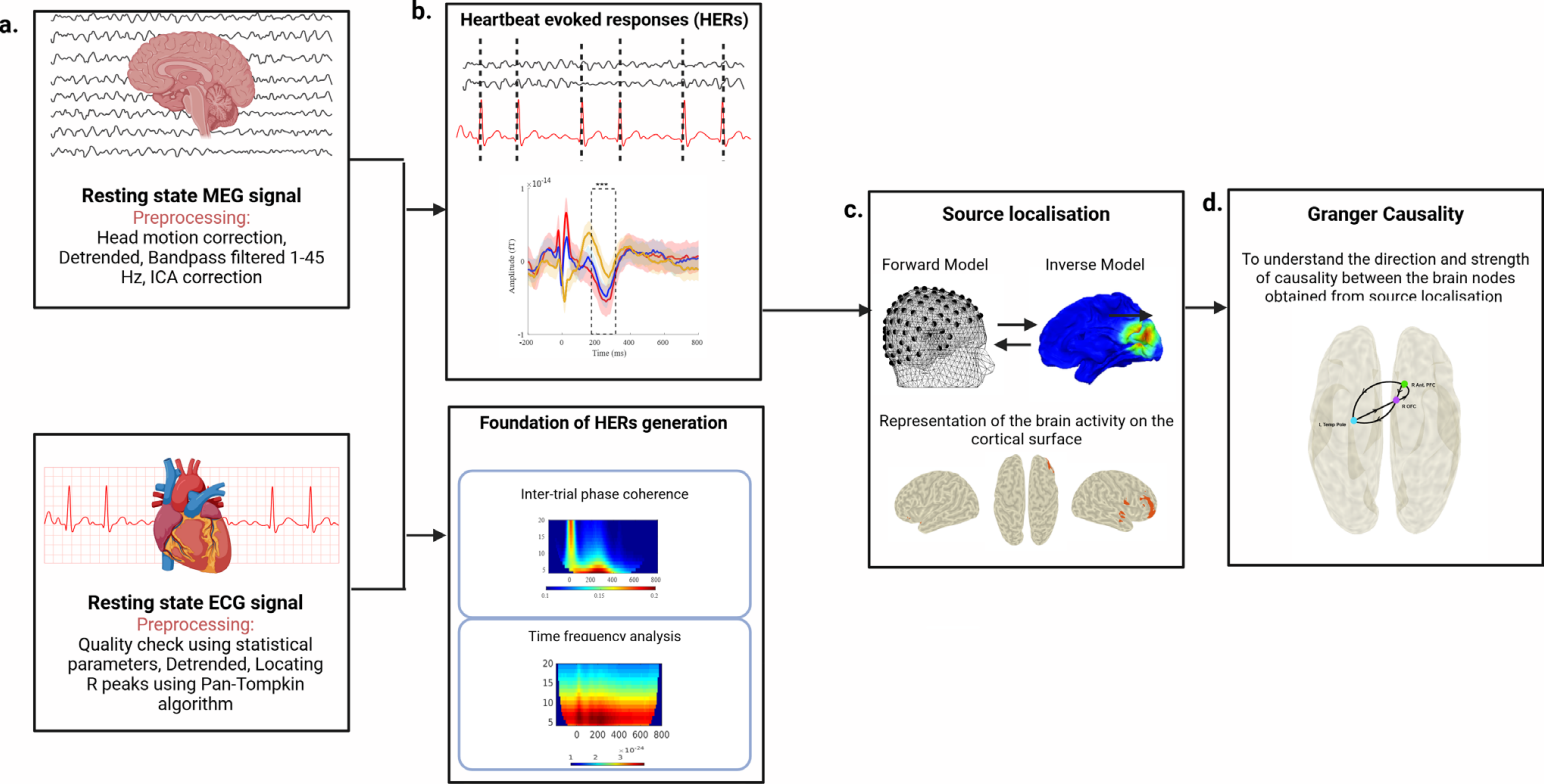
Overview of the Data Pre-processing and Analysis Pipeline. (a) Simultaneous MEG-ECG recordings undergo a preprocessing pipeline including detrending, bandpass filter and artifact removal using Independent Component Analysis (ICA). R peaks are detected from the electrocardiogram (ECG) waveform using Pan-Tompkin algorithm, serving as a trigger point to compute heartbeat evoked responses (HERs). (b) HERs are computed across all the age-groups (young, middle and old). Additionally, wavelet analysis is employed to investigate the mechanisms underlying HERs generation. (c) Source localization using eLORETA identifies cortical sources underlying HERs. (d) Finally, Granger causality maps casual functional relationships among cortical sources of HERs.

The ECG time series was detrended before passing it through the Pan-Tompkins algorithm (described in the Section 2) to locate R-peaks in the ECG waveform. Participants were further screened on the basis of their quality of EG signals. These signals were used to calculate parameters like variability of R-R interval, relative power of the QRS complex, skewness, and kurtosis (Supplementary Table S4). Following the screening, the analysis pipeline was performed on the 620 participants (Fig. 1).

### 2.4 Data analysis

All the data analysis was conducted in MATLAB using custom-made scripts. First, the fieldtrip toolbox (https://www.fieldtriptoolbox.org/) was used to load the data in the ‘.fif’ format (Oostenveld et al., 2010). The time series corresponds to the 309 x T where 309 are the total number of channels consisting of 102 magnetometers, 204 gradiometers, EOG, ECG, and other auxiliary electrodes. For the present study, 102 magnetometers were utilized from the MEG setup and a bipolar ECG electrode. The analysis pipeline is provided in Figure 1.

### 2.5 Pan-Tompkins algorithm

The cardiac cycle consists of distinct peaks of P wave, QRS complex, and T wave representing mechanical events in the cardiac muscles such as atrial contraction, ventricular contraction, and ventricular relaxation respectively. Pan-Tompkins algorithm was employed to locate the QRS complex, the most prominent peak in the ECG waveform (Sedghamiz, 2014). The vector representing the time points of the ECG waveform was passed into the algorithm which involves several steps including bandpass filtering the ECG time series generally in the range of 5–15 Hz, squaring the time series, applying a derivative filter and finally identifying the location of QRS complex using the moving window approach. The algorithm can be found at https://in.mathworks.com/matlabcentral/fileexchange/45840-complete-pan-tompkins-implementation-ecg-qrs-detector

### 2.6 Cardiovascular scores

To characterize age-related changes in baseline cardiovascular function and provide physiological context for the heart-brain analyses, cardiovascular measures including inter-beat interval (IBI), heart rate variability (HRV), heart rate (HR), systolic blood pressure (SBP), and diastolic blood pressure (DBP) were quantified for each participant. These measures were analyzed to examine their relationship with age and were not used as covariates in the HER or connectivity analyses. The inter-beat interval (IBI) and heart rate variability (HRV) were determined by analyzing ECG signals. The R peaks, identified using the Pan-Tompkins algorithm (refer to Section 2.5), were used to extract the IBI, representing the time difference between consecutive R peaks. The average IBI obtained was further used to calculate the heart rate (HR): the number of heartbeats per unit time (beats/min). HRV was computed by calculating the standard deviation of the IBI, which represents the fluctuations in the time interval between consecutive heartbeats. IBI, HRV, and HR were computed from the first 60 seconds of the resting-state ECG recording. Although longer recordings are commonly recommended for comprehensive cardiovascular assessment, previous validation studies have shown that ultra-short ECG recordings (30–120 seconds) provide reliable estimates of time-domain cardiovascular measures, particularly HRV derived from resting-state recordings. Accordingly, a 60-second window was used to obtain comparable baseline cardiovascular measures across the cohort (Lehavi et al., 2019; Munoz et al., 2015) which also optimized computational time. For each participant, cam-CAN recorded average systolic and diastolic pressure (recorded three times). To investigate the relationship between these cardiovascular scores and age, linear regression analysis was performed.

### 2.7 Heartbeat evoked responses (HERs)

To map the brain response time-locked to QRS peaks of ECG, the QRS peaks obtained from the Pan-Tompkins algorithm were used as trigger points to epoch the MEG time series. This allowed to test the key hypothesis of whether interoceptive signals act as the stimulant to the ongoing brain dynamics. Based on visual inspection, a pre-stimulus period of 200 ms (prior to QRS peak) and a post-stimulus of 800 ms (post to QRS peak) was identified that could capture uniquely one complete cardiac cycle. HERs were computed using averaging across trials and channels.

For continuous analysis, the aforementioned HERs was averaged across the number of channels and epochs resulting in a 1 x 1000 dimensional vector where 1000 represents the time points. The amplitude values between 180–320 ms post R peak were extracted in every subject. This time window was selected a priori based on previous HER studies demonstrating that the principal cortical response to cardiac afferent signals consistently occurs within this latency range, while remaining relatively unaffected by the cardiac field artifact (Kamp et al., 2021; Kim & Jeong, 2019; Park et al., 2018). To normalize the data, peak-to-peak subtraction was performed by subtracting the maximum amplitude value from each amplitude value for each participant. For categorical analysis, the matrix was stacked according to the age group i.e., younger adults, middle adults, and older adults. The global HERs were obtained by averaging the matrix across channels and trials.

A surrogate analysis as performed to validate the presence of HERs obtained from time locking to R peak of the ECG waveform. This involved computing shuffled HERs subject wise by randomly selecting non-R peaks to time lock essentially, random points from ECG time series excluding the R peaks, while maintaining the physiological IBI.

### 2.8 Assessment of the mechanism underlying HERs

#### 2.8.1 Inter-trial phase coherence (ITPC)

ITPC analysis (Oostenveld et al., 2010) was performed on both the HERs time series synchronized with the R peak and the shuffled HERs time series time-locked to random points in ECG. Power and phases were calculated using wavelet transformation in the frequency range of 4 to 20 Hz for all 102 magnetometers. ITPC was implemented using *ft_freqanalysis* in MATLAB (Oostenveld et al., 2010). The ITPC values are computed using:

$$ITPC_{f}=\left\lceil \frac{1}{n}\sum_{r=1}^{n}e^{i\theta }\right\rceil$$ (1)

wherein f derived is the frequency range at which the ITPC was calculated, $\frac{1}{n}\sum_{r=1}^{n}\left(\right)$ represents an average of complex vector over n trials; $e^{i\theta }$ is the Euler’s formula where θ is phase angle derived from complex Fourier coefficients. The value of ITPC ranges between 0 and 1, with higher values of ITPC corresponding to higher phase consistency across trials. As ITPC values exhibit significant variations based on the number of trials or participants, for group analysis consistency, randomly 177 participants were selected (N of the lowly populated group, Table 1) within each group (Van Diepen & Mazaheri, 2018). To evaluate the significance of phase clustering, the ITPC values obtained were contrasted with a critical value, computed using a significance level of p = 0.05. The critical threshold associated with the p-value was determined as per the formula:

$$ITPC_{crit}=\sqrt{-log\left(p\right)*n^{-1}}$$ (2)

where n represents the total count of trials. ITPC values surpassing this threshold were regarded as statistically significant.

#### 2.8.2 Time frequency estimation

To delve deeper into understanding the generation of HERs, the power distribution of HERs time series time-locked to R peaks and shuffled HERs time locked to random peaks were examined. The time-frequency analysis was performed by utilizing wavelet transformation with the *ft_freqanalysis* function from fieldtrip toolbox in MATLAB (Oostenveld et al., 2010). Furthermore, cluster permutation test was employed to find the significant clusters from the time frequency analysis, obtained from two conditions, that is, time locked to R peak and activity locked to shuffled R peak using custom-built MATLAB code. 1000 iterations of trial randomization were carried out to generate the permutation distribution at every frequency. A two-tailed test with a threshold of 0.05 was used to evaluate the time points that exhibit a significant difference in power.

In Section 2.8, all the analysis was performed for the 4 to 20 Hz frequency range. This was motivated by earlier findings (Kern et al., 2013) reporting the presence of pulsating artifacts in frequencies below 4 Hz, often attributed to pulsating blood vessels. Our analysis was restricted to the 4–20 Hz frequency range to avoid potential artifacts.

### 2.9 Source localization of HERs

Source localization was performed to obtain the generators of HERs in the brain averaged across all the participants using a current density technique known as exact low-resolution brain electromagnetic tomography (eLORETA), which was implemented using the MATLAB-based fieldtrip toolbox (Oostenveld et al., 2010). The origin (0,0,0) of the colin27 (Holmes et al., 1998) atlas was set to the anterior commissure, and fiducials were set accordingly before generating the head model. Using the single shell method (commonly used for MEG), the brain was segmented into a mesh/grid and employing channel position, the leadfield matrix was computed using *ft_prepare_leadfield.* The regularization parameter (l) was set at 0.1 for the localization of brain sources of HERs. Source power at grid locations (32480 grid points) was calculated using *ft_sourceanalysis* on the HERs time series occurring 180–320 ms post R peak for each age group. The HERs activity was normalized against the baseline activity between interval ([-200 -60]) time locked to R peak of the ECG waveform. Source power was thresholded at 95^th^ percentile, and regions exceeding this threshold were identified as significant sources of activation. These activations were then parcellated using the AAL atlas and visualized with MNE-Python. We have localized the sources for both categorical as well as continuous analysis where we have grouped the participants into three categories (18–40, 41–60, 61–88) and bins of 5 years from 18 to 88 years. For subsequent source time series reconstruction and GC analysis, a total 16 brain nodes were considered, combining sources from the continuous analysis.

### 2.10 Source time series reconstruction

Time series originating from the significant brain sources were reconstructed, participant-by-participant. This was achieved by multiplying the spatial filter generated from the statistically significant grid points for all the 620 participants to the pre-processed HERs time series (channel x time x trials). A consistent headmodel and electrode placement was used across all the participants. Applying the filter to the HERs epoched time series generated three source dipole time series, oriented along the x, y, and z axes. Due to the complexity introduced by three dipole orientations, the time series were projected onto the direction of the strongest dipole. This projection was determined by identifying the dominant eigenvector using singular value decomposition (SVD). Further, to simplify the analysis to estimate GC among few nodes, we applied a k-means clustering approach to classify significant grid points into significant clusters. Sources were grouped into 3 clusters. The centroid of each cluster was identified as a brain area using its proximity to the brain anatomical landmark following the MNI atlas. The time series reconstructed trial-wise for each node was the temporal coefficient projected on the first principal component of the dipolar time series obtained from the cluster. The reconstructed time series from each participant was treated as a trial, making a total of 177 trials for the young, 198 trials for the middle and 245 trials for the older age group. For a sanity check, HERs were computed for the reconstructed time series for all the significant brain parcels (Supplementary Fig. S1).

### 2.11 Granger causality

The statistical dependencies among the time series obtained at the source level were investigated by computing the directional interaction among the brain nodes of HERs and cardiac signals to understand the age associated changes associated with it. In this pursuit, conditional multivariate Granger Causality (MVGC) analysis was employed to elucidate the direction and magnitude of the causal influence (Dhamala et al., 2008; Granger, 1969) among corticocortical and cortico-cardiac interactions. In its most basic form, one can say that any random process, X, Granger causes another random process, Y, when X’s historical occurrences contribute to forecasting Y’s current values to a greater extent than what can be achieved by solely considering Y’s past events while conditioning out additional variables (Z). The GC analysis was conducted using the multivariate granger causality (MVGC) toolbox developed by (Barnett & Seth, 2014). The dataset consisted of the neural signals obtained as source time series from 16 brain nodes and ECG time series (average taken across all the epochs) for each participant. To meet the prerequisites of the GC algorithm, stationarity of these time series was assessed using the Augmented Dickey-Fuller test, employing the *‘adftest’* function in MATLAB. Since this revealed non-stationarity in the time series, a difference transformation to induce stationarity was applied, using MATLAB’s *diff* (first order) function. The vector autoregressive (VAR) model was utilized with a lag order of ‘p’, which was estimated by computing Akaike Information Criteria (AIC) using the function ‘*tsdata_to_infocrit’* in the MATLAB based MVGC toolbox (https://in.mathworks.com/matlabcentral/fileexchange/78727-the-multivariate-granger-causality-mvgc-toolbox). Various values of VAR order, p = 1, 2,……, 20 was tested. Based on the A/BIC criteria, a VAR order of 6 was chosen for GC estimation. The VAR model is considered robust for the given lag order ‘p’ when the Akaike Information Criterion (AIC) and Bayesian Information Criterion (BIC) attain their minimum values. This model can be described as (Barnett & Seth, 2014):

$$U_{t}=\left(\begin{array}{c} Xt\\ Yt\\ Zt \end{array}\right)$$ (3)

where $U_{t}$ consists of $X_{t},Y_{t},Z_{t}$ as the multivariate processes with this idea to eliminate any joint effect of Z on causality from Y to X. The VAR(p) decomposes as into regression coefficient and residuals covariance matrix. From this knowledge, two models, that is, reduced regression and full regression model, can be generated and demonstrated as:

Full regression model

$$X_{t}=\sum_{k=1}^{p}A_{xx}\cdot X_{t-k}+\sum_{k=1}^{p}A_{xy}\cdot Y_{t-k}+\sum_{k=1}^{p}A_{xy}\cdot Z_{t-k}+\varepsilon_{x,t}$$ (4)

Reduced regression model

$$X_{t}=\sum_{k=1}^{p}{A^{\prime }}_{xx}\cdot X_{t-k}+\sum_{k=1}^{p}A_{xy}\cdot Z_{t-k}+{\varepsilon^{\prime }}_{x,t}$$ (5)

Thus, the Granger causality from Y to X conditioned on Z is calculated by taking the log-likelihood ratio of the covariance residuals obtained from the full and reduced regression model. Mathematically, Granger causality is depicted as:

$$GC_{Y\to X\vee Z}\equiv ln\frac{{\Sigma^{\prime }}_{xx}}{\Sigma_{xx}\vee }$$ (6)

The significance of the GC was calculated against the null distribution generated using the source time series where the phases were staggered while keeping the amplitude conserved. GC peaks in the unshuffled data were considered statistically significant when the observed GC value reached beyond the 99^th^ percentile value of the null distribution. The multiple correction problem was handled by Bonferroni corrections.

### 2.12 Statistical analysis

#### 2.12.1 Effect of age and gender on HERs

To assess the impact of age on HERs, the time series was divided into three segments based on previous literature (Kamp et al., 2021): an early phase (180–320 ms post R peak), a middle phase (460–550 ms post R peak), and a late phase (650–750 ms post R peak). The *anovan* function in MATLAB was used for this analysis. A statistically significant difference between the age groups in the early phase window was observed as compared to the middle and late phase time windows. Furthermore, the impact of gender was examined along with age on HERs. As a result, the early phase HERs window, that is, 180–320 ms post R peak, was selected for further analysis. Refer to Supplementary Table S3 for details.

#### 2.12.2 Eliminating the confound of cardiac field artifact (CFA)

Given that the selected time window for HERs analysis encompasses the period of 180–320 ms after the R peak, which may be susceptible to contamination by cardiac field artifacts, measures were taken to address this confounding factor. To mitigate the influence of the cardiac field artifact, the ECG amplitude was extracted specifically within the time window of 180–320 ms following the R peak for each of the 620 participants. Subsequently, a linear regression analysis was performed between the mean ECG amplitude obtained from the 180–320 ms post R peak interval and the HERs obtained from the same interval. This regression analysis aimed to account for the ECG activity as a covariate, effectively eliminating its potential confounding effects on the HERs measurements within the specified time window (Supplementary Table S8).

#### 2.12.3 Linear regression

Linear regression analysis was performed separately for various parameters (magnitude obtained from peak-to-peak subtraction of HERs in the time interval of 180 to 320 ms post R peak, all the cardiovascular measures, estimated ITPC values, GC estimates) as the dependent variable while keeping age as the independent variable.

$$y=\beta_{0}+\beta_{1}*Age$$ (7)

*fitlm.m* function within MATLAB was utilized to perform a linear regression analysis which yielded F-statistics, regression coefficients and goodness of fit $R^{2}$ (Supplementary Tables S2, S8, S6). To assess the strength of the relationship between variables, the correlation coefficient was calculated using the built-in *corrcoef* function available in MATLAB.

## 3 Results

### 3.1 Physiological metrics of cardiovascular health across lifespan ageing

**Fig. 2.**
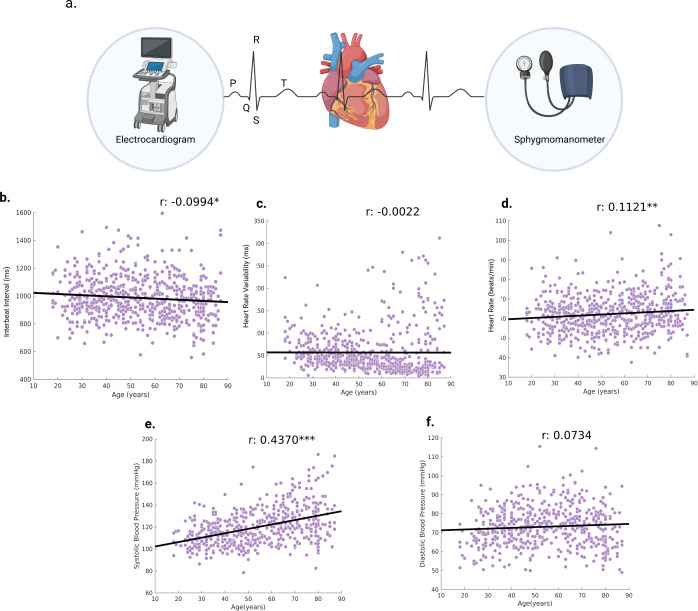
Cardiovascular measures across age. (a) Methods used to capture cardiac activity includes electrocardiogram (ECG) and sphygmomanometer which measures cardiovascular activities such as electric activity of the cardiac muscles and blood pressure respectively. Cardiovascular parameters include Inter beat interval (IBI), Heart rate variability (HRV), Heart rate (HR), Systolic and Diastolic blood pressure. (b) IBI significantly decreases (r = -0.0994; p = 0.0133). (c) HRV doesn’t change (r = -0.0022; p = 0.9573). (d) HR significantly increases (r = 0.1121; p = 0.0052). (e) Systolic blood pressure significantly increases (r = 0.4370; p < 0.001) and (f) Diastolic blood pressure remains unchanged (r = 0.0734; p = 0.0910) across age.

The pipeline presented in Figure 1 was used to analyze the electrocardiographic (ECG) and magnetoencephalographic (MEG) data from the Cam-CAN cohort (Shafto et al., 2014), comprising 620 participants aged 18 to 88 years. To assess the cardiovascular health, electrocardiographic parameters were utilized including inter-beat interval (IBI), heart rate variability (HRV), heart rate (HR), and blood pressure (BP) indicators such as systolic and diastolic pressure (Fig. 2a).

These parameters were derived from ECG time series and blood pressure scores provided by Cam-CAN. IBI significantly decreases (r = -0.0994, p = 0.0133; Fig. 2b) and HR significantly increases across age (r = 0.1121, p = 0.0052; Fig. 2d) (p < 0.05). However, HRV does not decrease significantly across age (r = -0.0022, p = 0.9573; Fig. 2c). In case of the trend of blood pressure scores across age, systolic pressure was significantly increasing (r = 0.4370, p < 0.001; Fig. 2e) whereas diastolic pressure was significantly increasing (r = 0.0734, p = 0.0910; Fig. 2f). Refer to the Supplementary Table S1 for additional details regarding the model.

### 3.2 Heartbeat evoked responses (HERs) across lifespan

**Fig. 3.**
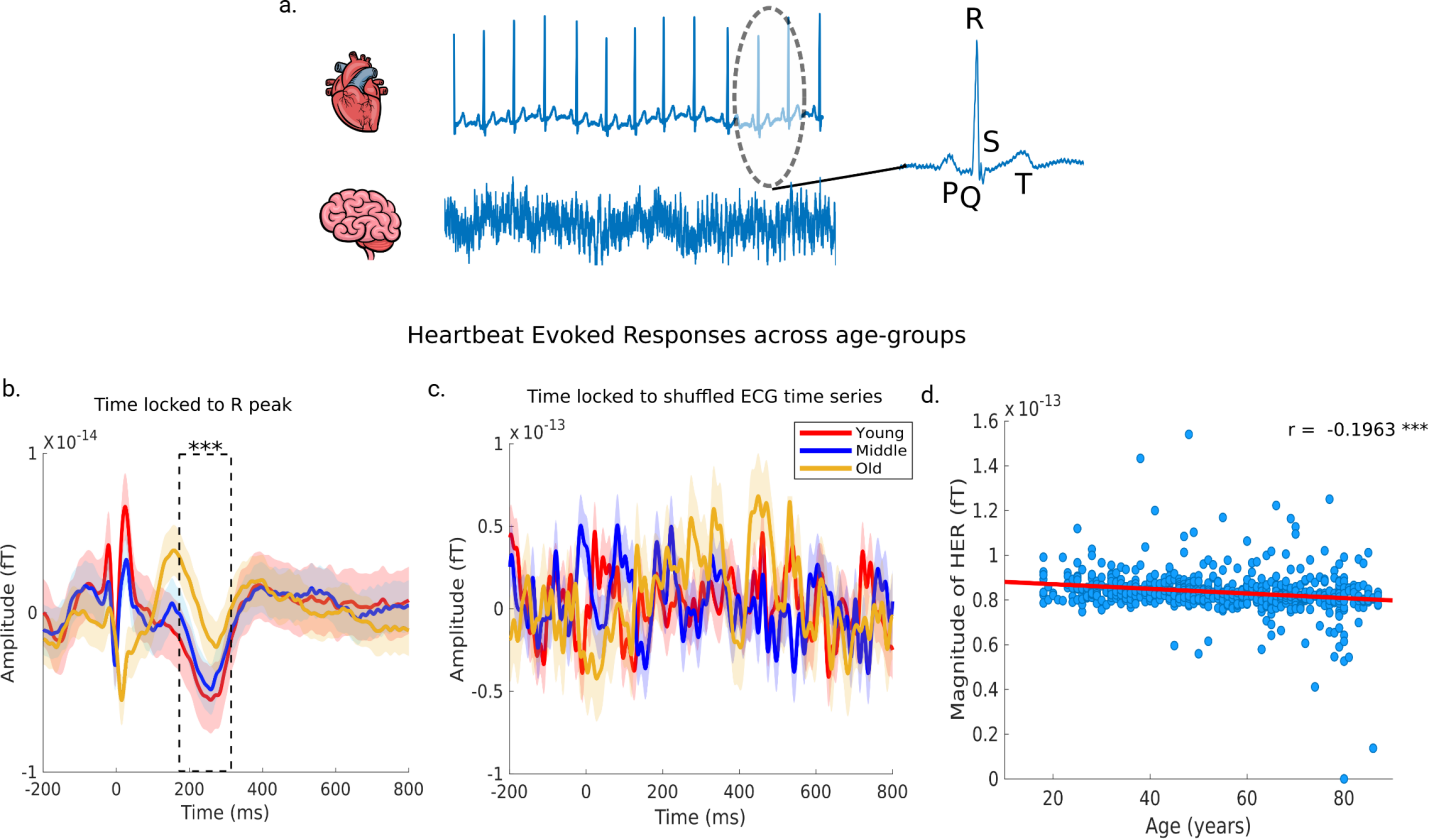
Heartbeat evoked responses (HERs) across age and gender**.** (a) Method: Simultaneous MEG-ECG recording: using R peak of the ECG waveform as the time points to epoch neural time series to obtain the HERs. (b) Group-wise analysis of HERs obtained from the time locked MEG activity to R peak of the ECG waveform. Statistical test conducted indicates that the HERs differences across the age groups are significant shown by the asterisks (F(2,619) = 9.48; p < 0.001). (c) Group wise analysis of HERs obtained from time locked activity to the shuffle ECG time series (sanity check). (d) Linear regression conducted between magnitude (obtained from peak to peak subtraction) of HERs and age (r = -0.1963; p < 0.001)

Simultaneous recordings of ECG and MEG were considered to create epochs of cardiac cycle comprising a QRS waveforms of 1 second duration, with first 200 ms representing pre-R peak period followed by 800 ms post R peak (Fig. 3a). The epochs were averaged to compute the HERs channel-by-channel. The global HERs calculated for three age groups, that is, young (18–40 years), middle (41–60 years), and old (61–87 years) (Fig. 3a), as well as for genders groups, that is, male and female (Fig. 3e). The main effect of age on HERs (F (2,619) = 9.47, p = 0.0001) was significant while the effect of gender became non-significant (F(1,619) = 0, p = 0.9631) in the early phase (180–320 ms post R peak) window. Furthermore, an interaction of both factors was found to be non-significant (F (2,619) = 0.33, p = 0.7213). Refer to Supplementary Table S2 for further details. To validate this result, the HERs were calculated with respect to the shuffled/ surrogate ECG time series (see Methods for detail) and, no time-locked activity was observed relative to the non-R peak points (Fig. 3c). Additionally, source-level HERs for the 16 significant nodes were calculated: Frontal Med Orb L (r = -0.2253, p < 0.0001), Frontal Mid Orb L (r = -0.2177, p < 0.0001), Frontal Sup Orb L (r = -0.2216, p < 0.001), Frontal Inf Orb L (r = -0.2387, p < 0.001), Frontal Inf Orb R (r = -0.1811, p < 0.001), Frontal Sup Orb R (r = -0.2003, p < 0.001), Frontal Mid Orb R (r = -0.1766, p < 0.001), Frontal Inf Tri L (r = -0.2085, p < 0.001), Frontal Med Orb R (r = -0.2178, p < 0.001), Frontal Sup R (r = -0.1766, p < 0.001), Frontal Sup Medial R (r = -0.2021, p < 0.001), Temporal Pole Mid R (r = -0.1805, p < 0.001), Temporal Pole Sup L (r = -.2296, p < 0.001), Temporal Pole Sup R (r = -0.1774, p < 0.001), Cingulum Ant R (r = -0.2188, p < 0.001), and Insula R (r = -0.1847, p < 0.001) showed significant correlation between the amplitudes extracted from the 180–380 ms post R peak window and age (Fig. 5h-w).

Further, linear regression analysis shows that the HERs amplitude extracted from the window of 180–320 ms post R peak of the ECG waveform corresponding to the window reaching the cortex significantly decreases across age (r = -0.1963, p < 0.001; Fig. 3d). Furthermore, when analyzed separately by the gender, this age-related decline in HERs amplitude remained significant for both male (r = -0.2161; p < 0.001) and female (r = -0.1735; p < 0.01) (Supplementary Fig. S1). Refer to Supplementary Table S3 for specifications of the regression model.

### 3.3 Relationship of cardiac rhythms and ongoing neural oscillations

**Fig. 4.**
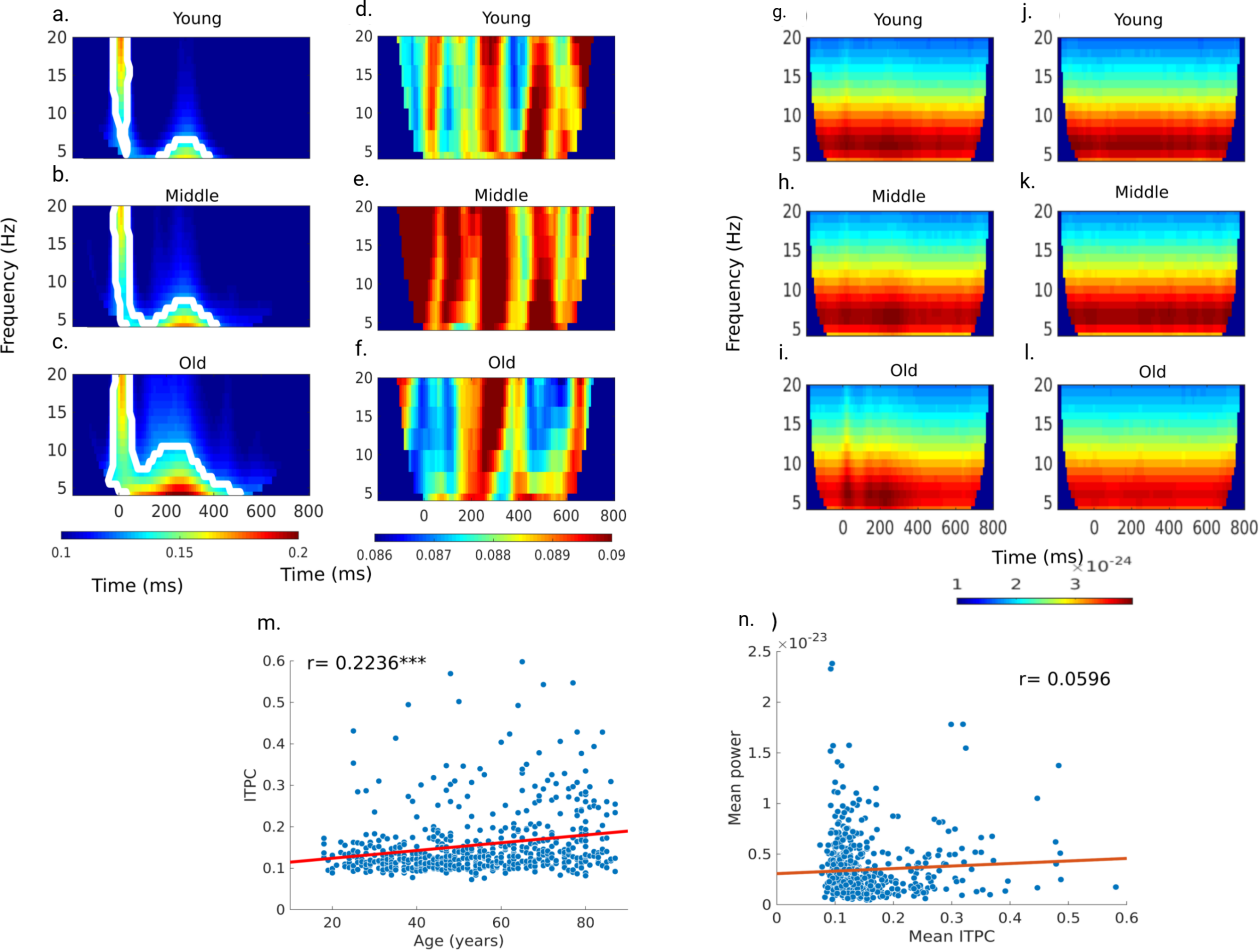
Mechanism of Heartbeat Evoked Responses**.** Inter trial phase coherence (ITPC) was computed on evoked data time locked to (a-c). R peak (d-f). shuffled R peak of ECG waveform calculated across all the age group. ITPC values within the white boundary are considered to be statistically significant, using critical value at p = 0.05. Time frequency spectrograms calculated on evoked data time locked to (g-i). R peak (j-l). shuffled R peak of ECG waveform (found no significant difference between the age groups; cluster permutation test at p = 0.05). (m) ITPC value (extracted from each subject) in theta band significantly increases across age (r = 0.2236; p < 0.001). (n) No correlation was observed between mean ITPC values and mean power extracted at around 180–320 ms post R peak of ECG waveform for each participant (r = 0.0596; p = 0.1382).

To substantiate the hypothesis that cardiac rhythms induce patterned changes in neural oscillations via phase resetting rather than a mere additive enhancement in spectral power (Park et al., 2018), the measure of inter-trial phase coherence (ITPC, see Section 2 for details) was employed, which increases if phase resetting occurs. Our findings indicate a significant increase in ITPC during the time interval of 180–320 ms following the R peak across all age groups (Fig. 4a-c: critical value of ITPC computed at significance level of p = 0.05). However, no time-locked activity was observed ITPC was examined relative to the shuffled ECG time series (preserving the physiological IBI) (Fig. 4d-f). Furthermore, predominant resetting of phase was observed within theta frequency band among young and middle age-group participants.

However, for the older age group, notable activity was observed in the alpha frequency band in addition to the activity in the theta band. The power analysis in the time-frequency domain showed no significant enhanced synchronization time locked to the R peaks (Fig. 4g-i) either for shuffled R peaks (Fig. 4j-l) of the ECG waveform (two-tailed cluster permutation test at p = 0.05). Interestingly, a significant increase in the ITPC values was observed across age in the theta frequency band (r = 0.2236; p < 0.001) (Fig. 4m). No significant correlation was observed between mean ITPC values and mean power extracted at around 180–320 ms post R peak of the ECG waveform (Supplementary Table S5; r = 0.0596; p = 0.1382) from each participant (Fig. 4n). No significant ITPC was observed in beta and gamma band in all age groups.

### 3.4 Organization of cortical sources of HERs

**Fig. 5.**
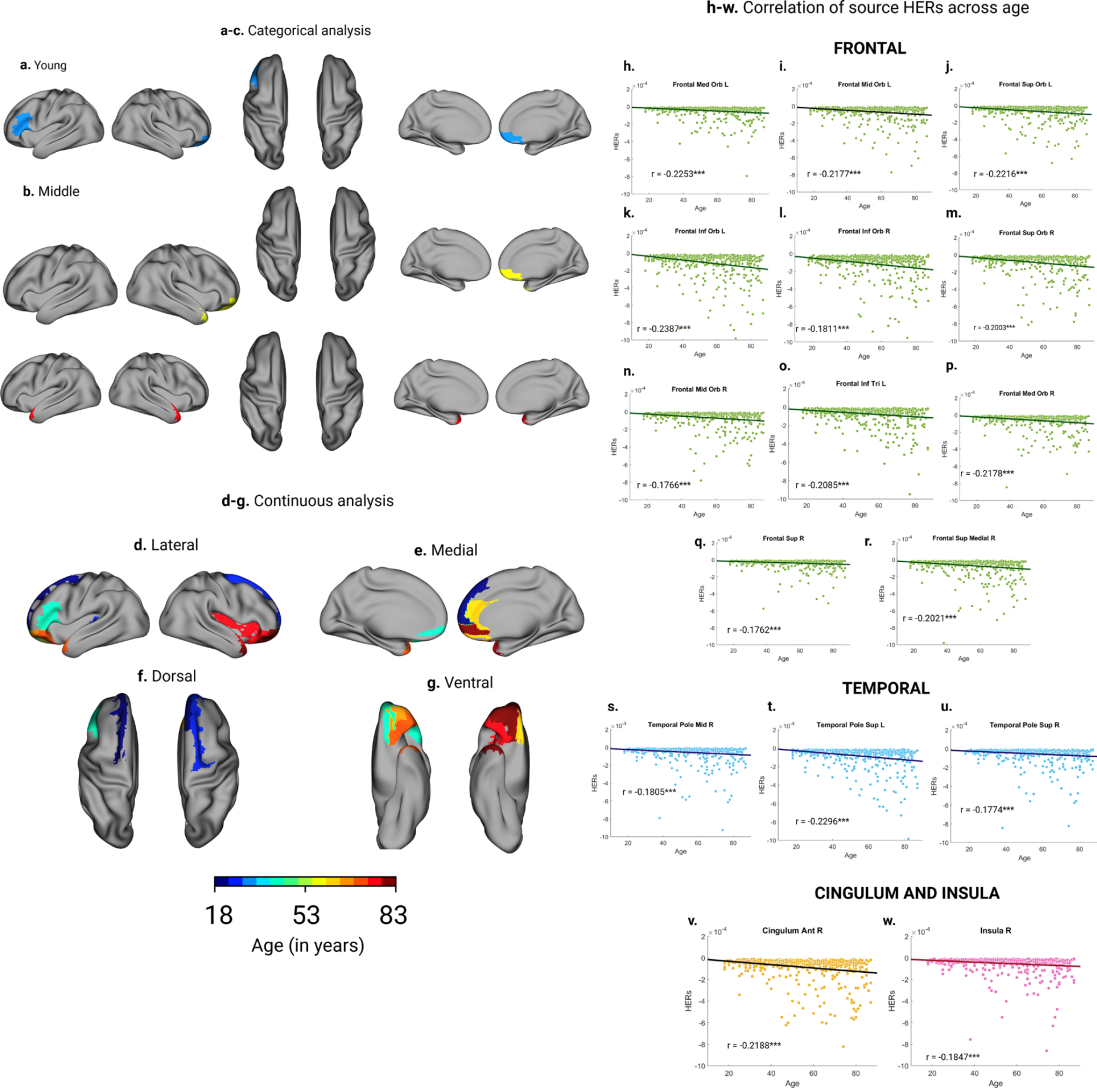
Cortical sources of Heartbeat evoked responses (HERs) across age: Continuous and categorical analysis. The sources represent the cortical areas involved in the processing of heartbeat which includes frontal-temporal regions across age. Panel (a-c) represent sources derived from categorical analysis, while panels (d-g) show results from continuous analysis. The statistical threshold was set at 95^th^ percentile and the source powers of grid points to surpassing this threshold were considered as significant sources of activation. All regions were approximated to the nearest Broadmann areas of the human brain. Regression between source-level HERs and age for different regions are (h-r) Frontal, (s-u) Temporal and (v-w) Cingulum and insula.

Exact low-resolution electromagnetic tomography (eLORETA) (see Methods for details of implementation) was employed to estimate the underlying sources contributing to HERs observed at the sensor level within the 180–320 ms window post R peak of the ECG time series. After the source power was computed for each participant, a grand averaging of the source power was calculated across in participants within each age group (young, middle-aged and elderly) as a group-level analysis. All the brain sources were located in the fronto-temporal network (Table 2, Fig. 5). The cohort of 620 participants were further divided into the bins of 5 years (18–23, 24–28,29–33,34–38, 39–43, 44–48, 49–53, 54–58, 59–63, 64–68, 69–73, 74–78, 79–83, 84–88 years) for the continuous analysis (Supplementary Table S6). These sources obtained from the continuous analysis were further used to conduct the directed connectivity. Source power surpassing the 95th percentile threshold was considered as significant sources of the HERs in the resting state condition for each age group. The sources obtained by both categorical and continuous analysis was plotted on the glass brain using MNE-Python (Gramfort et al., 2013). Additionally, all significant sources identified through the continuous analysis were aggregated to compute Granger causality.

**Table 2.** Anatomical labels (according to AAL parcellation) of cortical sources underlying HERs across lifespan.

Young (N = 177)	Middle (N = 198)	Old (N = 298)
Right superior orbitofrontal gyrus	Right superior orbitofrontal gyrus	Left superior temporal gyrus
Right middle orbitofrontal gyrus	Right middle orbitofrontal gyrus	Right superior temporal gyrus
Left inferior frontal gyrus	Right middle temporal gyrus	Left middle temporal pole
Right middle orbitofrontal gyrus	Right middle orbitofrontal gyrus	Right middle temporal pole

### 3.5 Directed functional connectivity between cortico-cortical and cortico-cardiac nodes

**Fig. 6.**
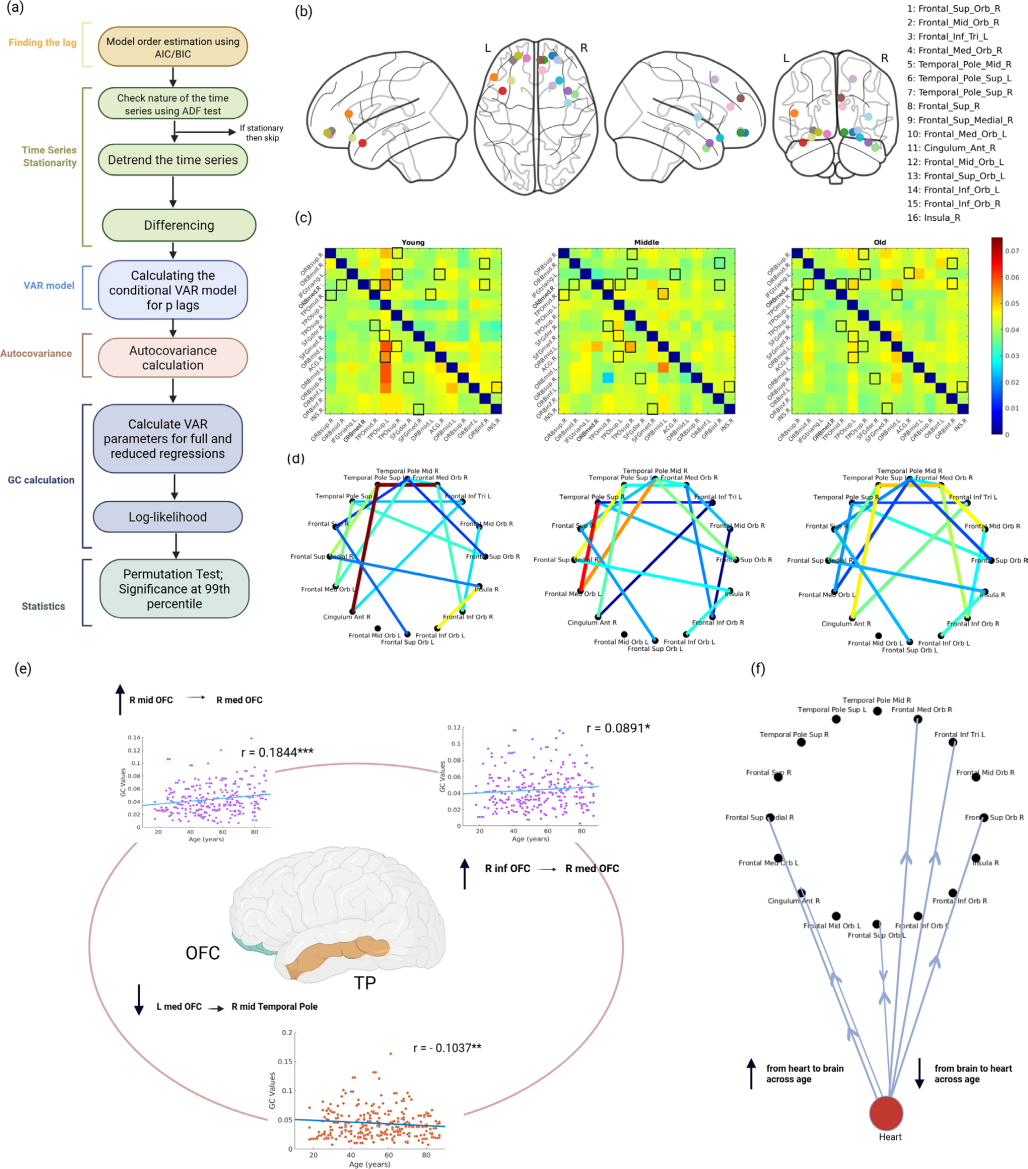
Cortico-cortical and cortico-cardiac information flow across lifespan**.** (a) Workflow used for the calculation of Granger Causality (GC) to evaluate information flow from brain-brain and brain-body interactions. (b) Visualization of brain nodes on a glass brain template, used for subsequent cortico-cortical connectivity calculation. (c) GC heatmaps showing connectivity between brain nodes; red indicated high GC value while blue indicates lower GC values. Black boxes highlight statistically significant values after permutation testing followed by Bonferroni correction. (d) Connectivity maps displaying significant connection between brain nodes (red line: strong connectivity; blue line: weak connectivity). (e) Age-related trends in connectivity between and within OFC and TP; connectivity from R mid OFC (r = 0.1846; p < 0.0001) and R inf OFC (r = 0.0891; p = 0.0269) increases to R med OFC across age; the connectivity from L mid OFC (r = -0.1037; p = 0.0099) to R mid TP decreases across age. (f) Cortico-cardiac connectivity: connection from the heart to brain increases across lifespan mostly, while connectivity from brain to heart decreases across age.

Conditional Multivariate Granger causality was computed across all possible HER sources and heart to assess source-level directed connectivity, utilizing the analysis pipeline using Figure 6a. This analysis provided insights into age-related changes in network dynamics. Heatmaps in Figure 6c. show GC values between all possible source pairs which are highlighted as nodes in Figure 6b. Figure 6d shows the connectivity between these nodes, depicting the strength of GC across different network connections. Notably, several nodes exhibited changes in GC patterns across age, as shown in Figure 6e. Additionally, Figure 6f demonstrates the connectivity between the heart and brain. Significant causal flow was summarized in Table 3a and Table 3b with the corresponding GC values provided for categorical analysis and regression coefficient for variations with age as a continuous variable. There was a clear increase in strength of causal information flow from heart to brain for all HER sources except left IFG. Interestingly, brain to heart interactions decrease over age. For further details on the casual flow transfer, refer to Supplementary Table S7.

**Table 3a.** Pairwise list of causally interacting cortical source pairs, showing age-related significant variations in causal strengths (GC values) in different age groups (categorical analysis) and regression coefficients (continuous analysis).

		Global GC values (10^-2^)				
From	To	Young	Middle	Old	Regression across age (r)	p-value
R mid OFC	R med OFC	4.04	4.20	4.78	0.1844	<0.0001
R inf OFC	R med OFC	4.40	4.97	3.98	0.0891	0.0269
L med OFC	R mid Temporal Pole	4.42	4.10	4.64	-0.1037	0.0099

**Table 3b.** Pairwise list of causally interacting cortico-cardiac pairs, showing age-related significant variations in causal strengths (GC values) in different age groups (categorical analysis) and regression coefficients (continuous analysis).

		Global GC values (10^-2^)				
From	To	Young	Middle	Old	Regression across age (r)	p-value
Cardiac	R superior OFC	6.26	5.51	7.2	0.1181	0.0033
	L inferior Frontal Gyrus	6.41	5.25	5.54	-0.1099	0.0063
	R middle OFC	5.95	5.87	7.51	0.1637	<0.001
	R superior frontal gyrus	5.39	5.59	6.54	0.1377	0.0006
	R anterior cingulate gyrus	5.70	5.75	7.34	0.1701	<0.001
	R superior OFC	6.13	5.50	6.70	0.0978	0.0151
R superior temporal pole	Cardiac	4.94	4.70	4.06	-0.1212	0.0026
R superior OFC		4.73	5.25	4.04	-0.0915	0.0230

## 4 Discussion

The present study characterizes the age-associated changes in the cortical representation of the cardiac signals: Heartbeat evoked responses (HERs), neural mechanisms underlying the generation of HERs and corresponding neural networks using a large cross-sectional resting state dataset (N = 620). Four key empirical observations emerged from the subsequent investigations. *First,* time-locked HERs over a temporal window of 180–320 ms was observed post R peak of the ECG waveform (Fig. 3) which was consistent for all the participants. HER amplitude in these temporal boundaries decreased across age gradually, establishing the normative patterns of age-related alteration in the processing of cardiac rhythm. *Second*, the mechanism leading to the generation of HER was identified, essentially a phase reset of the spontaneous brain oscillations triggered by the ECG waveform rather than additive spectral power enhancement in theta band (4–8 Hz) frequency band, another alternative that could have been a potential underlying mechanism (Fig. 4). Further, a linear increasing trend of the ITPC values across age was observed in the theta (4–8 Hz) frequency band, suggesting that aging is associated with more consistent phase alignment of neural responses to cardiac events. *Third*, the neural sources of HERs that were identified during the resting state are primarily located in fronto-temporal areas: right superior orbitofrontal, right middle frontal gyrus, left inferior frontal gyrus, right medial orbitofrontal gyrus, right middle temporal pole, left superior temporal pole, right superior temporal pole, and left middle temporal pole. Furthermore, a clear pattern of sources shifting from frontal to temporal regions as a function of the age was also identified (Fig. 5). *Fourth*, directed connectivity analysis revealed age-related reorganization of heart-brain interactions, with heart-to-brain Granger causality increasing and brain-to-heart Granger causality decreasing across the lifespan. Additionally, intra-orbitofrontal connectivity increased with age, suggesting age-related functional reorganization of networks involved in interoceptive and autonomic processing (Fig. 6).

Hence, the present findings firmly quantify the age associated alterations in the cortical representation of cardiac rhythms and circuit reorganization, to be the best of our knowledge, have not been previously reported. In the subsections, we delve into the implications of salient findings from our study.

### 4.1 Age-related changes in cortical processing of cardiac signals

The field of interoception faces key questions such as the nature of HER organization during resting state, their reorganization during healthy ageing in terms of temporal landmarks and correspondingly whether is a change in the underlying generative networks what implications might these changes have on cognition and emotion? HER amplitude is widely interpreted as an index of neurophysiological interoceptive processing, reflecting bottom-up cortical responses to cardiac input (Banellis & Cruse, 2020; Montoya et al., 1993; Pollatos & Schandry, 2004). Here, a time locked HER around 180–320 ms post R peak of the ECG waveform representing the time in which the cardiac information is relayed to the cortex and a notable decline in its amplitude across age was observed consistently from a categorical and continuous assessment of the data. Similarly, source level HERs showed a significant decline across age for frontal, temporal, cingulum and insular regions. Prior literature suggests that HER amplitude serves as an index of interoceptive processing (Coll et al., 2021), which is known to decline across the lifespan (S. Khalsa et al., 2009). While there are few speculations for this age-related reduction in interoceptive awareness might be brain undergoing structural changes, such as atrophy in white matter (Pathak et al., 2022), degradation of prefrontal cortex (Cabeza & Dennis, 2013; Zanto & Gazzaley, 2019), and vascular stiffening (Gauthier et al., 2013). More broadly, changes in interoceptive processing may contribute to differences in cognitive and behavioural functions across the lifespan, reflecting altered integration of bodily signals with ongoing neural activity.

### 4.2 Cardiac signal induces phase resetting on the spontaneous brain activity rather than the amplitude

While addressing how heartbeat influence the spontaneous brain activity, prior work (Park et al., 2018) suggests that heartbeat may act as an “internal trigger” and reset the phase of the spontaneous brain rhythms, rather than contributing to evoked responses furthermore connecting it to bodily self-consciousness. In the present study, a comparable phenomenon was observed: ITPC increases around 180 ms post R peak of the ECG waveform with no change in the spectral power. On shuffling the R peaks of ECG waveform, no time locked activity was observed. Interestingly, oscillations in theta frequency band for the young and the middle age group were strongly phase reset whereas in older participants, in addition to the theta, alpha band was also affected. In older adults, phase resetting extended beyond theta to include the alpha band (8–12 Hz). Given the established role of alpha oscillations in regulating cortical excitability and information gating, this broader frequency involvement may reflect a shift in how cardiac signals modulate large-scale neural dynamics (Sahoo et al., 2020; Thuwal et al., 2021). This could indicate either compensatory recruitment of additional oscillatory mechanisms or reduced specificity of phase-locking with age. Importantly, these findings support a phase-resetting mechanism underlying HER generation, while highlighting age-related changes in the frequency-specific organization of cardiac–brain coupling. However, the functional implications of these changes for cognition or behavior remain to be determined.

### 4.3 Fronto-temporal network reconfiguration in the representation of cardiac signals

The current findings further suggest that aging is not only associated with change in flexibility in the temporal alignment of interoceptive signals, but also with a broader reorganisation of the networks supporting their cortical representation. In particular, the continuous analysis revealed the gradual shift in the representation of HERs from frontal to temporal regions. With an advancing age, the prefrontal and orbitofrontal cortices may lose their capacity to flexibly integrate interoceptive information, likely reflecting underlying structural changes in these areas (Pathak et al., 2022; Zanto & Gazzaley, 2019). At the same time, the observed shift away from the orbitofrontal cortex which is increasingly recognized as a critical interoceptive hub alongside the insula (Elorette et al., 2021), may not reflect a complete loss, rather a reweighing of how cardiac signals are represented across lifespan (Romero Sosa et al., 2021). Such configuration could indicate that in older individuals, interoceptive signals become less embedded in executive processes and more grounded in affective and memory-related representations, consistent with a lifetime of accumulated experiences and reduced exposure to novelty. Taken together, these findings suggest that aging alters the integration of bodily signals across brain systems, reflecting a reorganization of functional networks rather than an uniform global decline in interoceptive processing.

### 4.4 Age-related dynamics of cortico-cortical and cortico-cardiac connectivity

With aging, large-scale cortical networks undergo reorganization (Sahoo et al., 2020; Thuwal et al., 2021) accompanied by changes in their interaction with cardiac signals. Cardiac afferent signals continuously convey information about the physiological state of the body to the central nervous system and contribute to cognitive and autonomic processes, whereas descending pathways regulate cardiac function to maintain homeostasis. In the present study, this bidirectional communication exhibited systematic age-related alterations, with heart-to-brain Granger causality increasing and brain-to-heart Granger causality decreasing across the adult lifespan.

Because Granger causality quantifies the extent to which past activity in one system improves the prediction of future activity in another (Barnett & Seth, 2014; Dhamala et al., 2008), the observed increase in heart-to-brain Granger causality suggests that the temporal structure of cardiac activity becomes increasingly informative for predicting subsequent neural activity with age. This interpretation is supported by the concurrent increase in heartbeat-related phase coherence, indicating that neural activity becomes more consistently aligned to recurring cardiac events.

One possible explanation is that aging is accompanied by a progressive reduction in physiological and neural complexity, resulting in less variable and more stereotyped temporal dynamics across interacting biological systems (Giuliani et al., 1998; Goldberger et al., 2002; Lipsitz et al., 1992; Manor & Lipsitz, 2013). Under this framework, ongoing cortical activity may become increasingly constrained by the recurring rhythm of the heartbeat, producing more consistent phase alignment across successive cardiac cycles. Consequently, cardiac activity becomes a more reliable predictor of subsequent neural activity, giving rise to the observed increase in heart-to-brain Granger causality. In contrast, the reduction in brain-to-heart Granger causality suggests that cortical activity becomes less predictive of subsequent cardiac dynamics with age. This finding may reflect reduced autonomic flexibility and diminished efficiency of top-down autonomic regulation (Wu et al., 2024) consistent with the age-related changes in cardiovascular measures observed in the present cohort.

At the network level, increased intra-orbitofrontal connectivity was also observed. Given the established role of the orbitofrontal cortex in autonomic and interoceptive processing, this finding may reflect age-related functional reorganization of heart-brain networks (Resnick et al., 2007; Romero Sosa et al., 2021). These mechanistic interpretations remain speculative because the current study did not directly assess physiological complexity, autonomic function, or interoceptive processing.

In summary, the present study demonstrates that aging is associated with systematic changes in the cortical representation of cardiac signals and in the dynamics of heart–brain interactions. Specifically, HER amplitude decreases with age, while phase consistency increases, suggesting alterations in how cardiac signals are temporally aligned with ongoing neural activity. In parallel, source-level analyses indicate a redistribution of activity across fronto-temporal networks, accompanied by changes in directed connectivity between cortical regions and the heart. Importantly, while these findings characterize neural and physiological changes in cardiac-brain coupling, their implications for cognition or subjective experience cannot be directly inferred in the absence of behavioral measures. Future studies integrating neural, physiological, and behavioral assessments will be critical to establish how these age-related changes in interoceptive processing relate to cognition and affect. A key limitation of the present study is the absence of direct measures of interoceptive awareness or accuracy, which constrains the interpretation of HER amplitude as a proxy for interoceptive ability. Addressing this limitation in future work will be essential for linking neural markers of cardiac processing to behaviour.

## Data and Code Availability

The neuroimaging data of healthy participants were obtained from publicly available CamCAN repository (available at http://www.mrc-cbu.cam.ac.uk/datasets/camcan). (Shafto et al., 2014; Taylor et al., 2017). Please note all data are publicly available after signing a non-disclosure clause with data provider (CamCAN). Custom code used for the analysis presented in this study can be downloaded from GitHub link https://github.com/kirtisaluja/HER-Across-Ageing/

## Supplementary Material

Supplementary Material
