# Supplementary material for: When the inner clock fades: Interoceptive decline and consolidation of phase resetting in cortical rhythms by cardiac events underlie healthy lifespan aging
Source: Imaging Neurosci (Camb). 2026 Jul 29;4:IMAG.a.1319. doi: 10.1162/IMAG.a.1319 (PMC13449928; doi:10.1162/IMAG.a.1319)

Supplementary Information

When the inner clock fades: Interoceptive decline and consolidation of phase resetting in cortical rhythms by cardiac events underlie healthy lifespan aging

Authors: Kirti Saluja[1], Dipanjan Roy[2], Arpan Banerjee[1]*

Affiliations:
[1]National Brain Research Centre, NH-8, Manesar, Haryana – 122052
[2] Centre for Brain Science and Applications (CBSA), School of AIDE, Indian Institute of Technology, Jodhpur - 342030
* Corresponding Author
Email : arpan@nbrc.ac.in

Supplementary Tables

Table S1: Regression table for cardiovascular measures with age. F value, β coefficient, goodness of fit, and significance of the model are reported.

Independent variable	Dependent variable	F-stats	1	R^2	p-value
Age	Interbeat interval (IBI)	6.16	-0.83443	0.00987	0.0133
	Heartbeat variability (HRV)	0.00286	-0.0056268	4.63e-^{06}	0.957
	Heart Rate (HR)	7.87	0.060119	0.0126	0.00518
	Systolic Blood Pressure	125	0.40098	0.191	3.56e^{-26}
	Diastolic Blood Pressure	2.87	0.041845	2.87	0.091

Table S2: Effect of age and topography on the Heart evoked responses (HERs) using ANOVA

S2.1. Two Way ANOVA and multiple comparison test applied on 180-320 ms post R peak

(a)

Source	SS	df	MS	F	Prob>F
Columns	1.44578e-27	2	7.22888e-28	54.87	9.30711e-20
Rows	2.8673e-26	101	2.83891e-28	21.55	1.5502e-70
Error	2.6615e-27	202	1.31757e-29		
Total	3.27803e-26	305			

(b)

Age group 1	Age group 2	Lower Limit	Estimate	Upper Limit	p-value
Young	Middle	-2.4024e-15	-1.2111e-15	-1.985e-17	0.045258
Young	Old	-6.2869e-15	-5.0957e-15	-3.9044e-15	9.5606e-10
Middle	Old	-5.0758e-15	-3.8846e-15	-2.6933e-15	9.5611e-10

S2.2. Two Way ANOVA and multiple comparison test applied on 460-550 ms post R peak

(a)

Source	SS	df	MS	F	Prob>F
Columns	4.55773e-29	2	2.27886e-29	1.55	0.2146
Rows	6.92583e-27	101	6.85736e-29	4.67	0
Error	2.96852e-27	202	1.46957e-29		
Total	9.94003e-27	305			

(b)

Age group 1	Age group 2	Lower Limit	Estimate	Upper Limit	p-value
Young	Middle	-1.8179e-15	-5.5983e-16	6.9826-16	0.54975
Young	Old	-2.1977e-15	-9.3961e-16	3.1848e-16	0.18661
Middle	Old	-1.6379e-15	-3.7978e-16	8.7831e-16	0.75906

S2.3. Two Way ANOVA and multiple comparison test applied on 650-750 ms post R peak

(a)

Source	SS	df	MS	F	Prob>F
Columns	5.25924e-30	2	2.62862e-30	1.76	0.1748
Rows	4.13579e-27	101	4.09484e-29	27.39	0
Error	3.0198e-28	202	1.49495e-30		
Total	4.44303e-27	305			

(b)

Age group 1	Age group 2	Lower Limit	Estimate	Upper Limit	p-value
Young	Middle	-1.2331e-16	2.7795e-16	6.7922e-16	0.23568

Young	Old	-4.0157e-16	-3.0375e-19	4.0096e-16	1
Middle	Old	-6.7952e-16	-2.7826e-16	1.2301e-16	0.23494

Table S3: Regression table for Heart evoked responses with age. F value, β coefficient, goodness of fit, and significance of the model are reported.

Independent variable	Dependent variable	F-stats	1	R^2	p-value
Age	Heart evoked responses (HERs) amplitude	24.8	-1.0356e-16	0.0386	$8.34e^{-07}$

Table S4: List of participants excluded from the study along with their exclusion criteria. The exclusion criteria based on ECG signal processing measure such as skewness, high R-R coefficient of variance, relative QRS complex power. Initial number of participants : **650,** Participants after exclusion : **620**

S.No.	Subject ID	Age	Exclusion Criteria
1	CC310160	41	ECG signal not available
2	CC420729	56	ECG signal not available
3	CC610050	71	ECG signal not available
4	CC610061	76	ECG signal not available
5	CC620044	73	ECG signal not available
6	CC620499	71	ECG signal not available
7	CC620557	74	ECG signal not available
8	CC710088	83	ECG signal not available
9	CC710154	83	ECG signal not available
10	CC711035	88	ECG signal not available
11	CC720071	82	ECG signal not available
12	CC720103	80	ECG signal not available
13	CC721519	79	ECG signal not available
14	CC722077	82	ECG signal not available
15	CC110045	24	High R-R coefficient of variance
16	CC121411	26	High R-R coefficient of variance
17	CC620152	73	High R-R coefficient of variance
18	CC720685	81	High R-R coefficient of variance
19	CC410121	52	High skewness and kurtosis value
20	CC420061	57	High skewness and kurtosis value
21	CC722542	79	High skewness and kurtosis value
22	CC410226	56	Relative power of the QRS complex is high due to a split in the R peak
23	CC420180	56	Relative power of the QRS complex is high due to a split in the R peak
24	CC610288	69	Relative power of the QRS complex is high due to a split in the R peak
25	CC610658	78	Relative power of the QRS complex is high dude to a split in the R peak
26	CC720646	81	Noisy signal
27	CC410094	54	Noisy trials (MEG)
28	CC510480	68	Noisy trials (MEG)

29	CC620490	74	Noisy trials (MEG)
30	CC620436	78	Noisy trials (MEG)

Table S5: Regression table for ITPC values and spectral power (conducted for theta frequency band). The ITPC and spectral power values extracted from the significant window (180-400 ms post R peak) and theta frequency range (4-7 Hz). F value, β coefficient, goodness of fit, and significance of the model are reported.

Independent variable	Dependent variable	F-stats	1	R^2	p-value
ITPC values	Spectral power values	2.2	0.059607	0.00355	0.138

Table S6: Cortical sources of Heartbeat evoked responses (HERs) across age : Continuous Analysis

S.No.	Age group	N	Brain Region
1	18-23	20	Frontal_Sup_R<br>Frontal_Sup_Orb_R<br>Frontal_Sup_Medial_R<br>Frontal_Med_Orb_R<br>Cingulum_Ant_R<br>Thalamus_L
2	24-28	39	Frontal_Sup_R<br>Frontal_Med_Orb_R<br>Frontal_Med_Orb_L<br>Rectus_R<br>Cingulum_Ant_R<br>Heschl_L
3	29-33	41	Frontal_Sup_Orb_R<br>Frontal_Mid_Orb_L<br>Frontal_Inf_Tri_L<br>Frontal_Med_Orb_L<br>Frontal_Med_Orb_R<br>Cingulum_Ant_R
4	34-38	55	Frontal_Sup_Orb_R<br>Frontal_Mid_Orb_R<br>Frontal_Med_Orb_R<br>Rectus_R<br>Amygdala_R<br>Putamen_R
5	39-43	47	Frontal_Sup_Orb_L<br>Frontal_Sup_Orb_R<br>Frontal_Mid_Orb_L<br>Frontal_Inf_Tri_L<br>Frontal_Inf_Orb_L<br>Frontal_Med_Orb_L
6	44-48	62	Frontal_Sup_Orb_R<br>Frontal_Mid_Orb_R<br>Frontal_Med_Orb_R<br>Rectus_R<br>Cingulum_Ant_R<br>Temporal_Pole_Mid_R
7	49-53	46	Hippocampus_R

			ParaHippocampal_R<br>Amygdala_R<br>Temporal_Pole_Mid_R<br>Cerebullum_3_R<br>Cerebullum_10_R
8	54-58	53	Frontal_Mid_Orb_L<br>Frontal_Inf_Orb_L<br>Temporal_Pole_Sup_L<br>Temporal_Pole_Mid_L<br>Temporal_Pole_Mid_R<br>Cerebellum_10_R
9	59-63	48	Frontal_Sup_Orb_R<br>Frontal_Mid_Orb_L<br>Frontal_Mid_Orb_R<br>Frontal_Med_Orb_R<br>Rectus_R<br>Cingulum_Ant_R
10	64-68	54	Frontal_Mid_Orb_L<br>Amygdala_L<br>Amygdala_R<br>Putamen_L<br>Putamen_R<br>Temporal_Pole_Mid_R
11	69-73	47	Frontal_Inf_Orb_L<br>Olfactory_L<br>Amygdala_L<br>Putamen_L<br>Temporal_Pole_Sup_L<br>Temporal_Pole_Mid_L
12	74-78	42	Frontal_Inf_Orb_R<br>Insula_R<br>Putamen_R<br>Pallidum_R<br>Temporal_Pole_Sup_R<br>Temporal_Pole_Mid_R
13	79-83	54	Frontal_Sup_Orb_R<br>Frontal_Mid_Orb_R<br>Frontal_Med_Orb_R<br>Rectus_R<br>Amygdala_R<br>Temporal_Pole_Mid_R
14	84-88	19	Vermis_3<br>Vermis_4_5<br>Vermis_6<br>Vermis_7<br>Vermis_8<br>Vermis_10

Table S7: Pairwise list of casually interacting cortico-cortical and cortico-cardiac pairs

(a) Significant causal interaction between the cortico-cortical nodes

From	To	Global GC values (10^{-2})
Frontal_Sup_Orb_R	Temporal_Pole_Mid_R	4.16
Frontal_Mid_Orb_R	Frontal_Med_Orb_R	4.38
Temporal_Pole_Mid_R	Frontal_Med_Orb_R	4.34
Temporal_Pole_Mid_R	Frontal_Sup_R	4.10
Temporal_Pole_Sup_L	Frontal_Med_Orb_R	4.87
Temporal_Pole_Sup_L	Frontal_Sup_Med_R	4.62
Temporal_Pole_Sup_L	Cingulum_Ant_R	4.88
Temporal_Pole_Sup_R	Frontal_Sup_Orb_R	4.33
Temporal_Pole_Sup_R	Frontal_Inf_Tri_L	4.01
Temporal_Pole_Sup_R	Frontal_Med_Orb_L	4.60
Frontal_Sup_R	Frontal_Sup_Orb_L	4.13
Frontal_Sup_Med_R	Insula_R	4.20
Frontal_Med_Orb_L	Temporal_Pole_Mid_R	4.42
Cingulum_Ant_R	Frontal_Inf_Tri_L	4.18
Frontal_Inf_Orb_R	Frontal_Mid_Orb_R	4.08
Frontal_Inf_Orb_R	Frontal_Med_Orb_R	4.40
Insula_R	Frontal_Inf_Orb_L	4.49

(b) Significant causal interaction between the cortico-cardiac nodes

From	To	Global GC values (10^{-2})
Frontal_Inf_Tri_L	Cardiac	4.79
Temporal_Pole_Mid_R		4.75
Temporal_Pole_Sup_L		4.74
Temporal_Pole_Sup_R		4.51
Frontal_Sup_R		4.74
Frontal_Sup_Med_R		4.52
Frontal_Med_Orb_L		4.83
Cingulum_Ant_R		4.61
Frontal_Mid_Orb_L		5.47
Frontal_Inf_Orb_L		5.12
Frontal_Inf_Orb_R		4.72

Insula_R		4.52
Cardiac	Frontal_Sup_Orb_R	6.39
	Frontal_Mid_Orb_R	5.87
	Frontal_Inf_Tri_L	5.69
	Frontal_Med_Orb_R	6.54
	Temporal_Pole_Mid_R	5.90
	Temporal_Pole_Sup_R	6.36
	Temporal_Pole_Sup_L	6.33
	Frontal_Sup_R	5.45
	Frontal_Sup_Medial_R	5.91
	Frontal_Med_Orb_L	6.27
	Cingulum_Ant_R	6.36
	Frontal_Mid_Orb_L	5.85
	Frontal_Sup_Orb_L	6.15
	Frontal_Inf_Orb_L	6.10
	Frontal_Inf_Orb_R	5.92
	Insula_R	5.76

Table S8: Regression table for ECG amplitude with HERs amplitude extracted from a time window of 180-320 ms post R peak. F value, β coefficient, goodness of fit, and significance of the model are reported.

Independent variable	Dependent variable	F-stats	1	R^2	p-value
ECG amplitude	Heart evoked responses (HERs) amplitude	3.19	-5.1195e-12	0.00513	0.0746

Supplementary Figure 1:

Group-wise analysis of HERs across gender i.e., time locked MEG activity to the R peak (represented by the dotted line) of the ECG waveform for two gender (male and female). (a) Statistical test conducted indicated that there is no significant difference between the two groups (F (1,619) = 0.02; p = 0.8801). The shaded area shows the standard deviation. Linear regression conducted magnitude (obtained from peak to peak subtraction) of HERs across age in b. Females (r= -0.2161; p<0.001) and c.. Males (r = -0.1735; p<0.01).

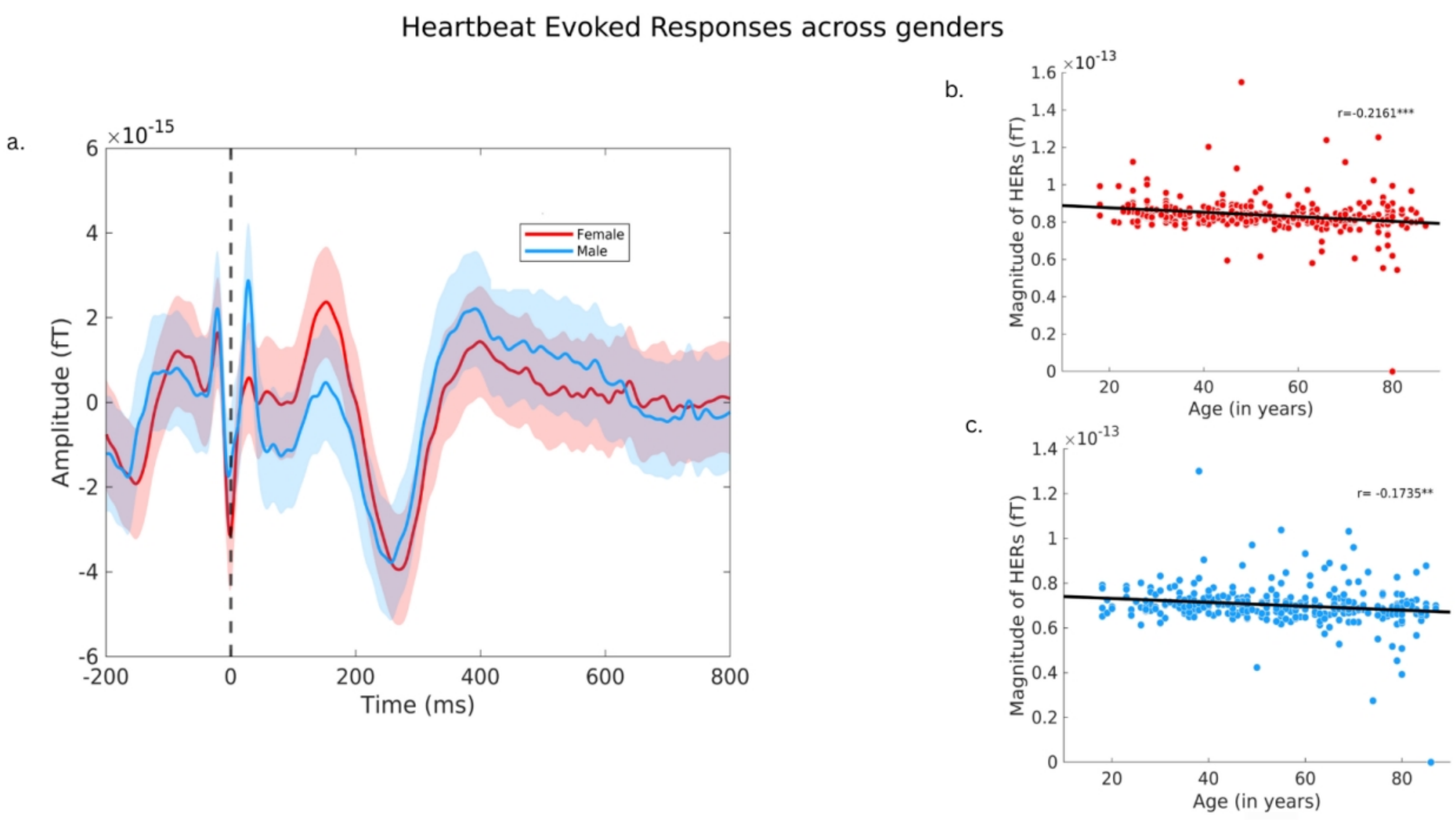

Supplement: Supplementary file 1 [file IMAG.a.1319_supp.pdf]
